# Comparative performance of modified full-length and truncated *Bacillus thuringiensis-cry1Ac* genes in transgenic tomato

**DOI:** 10.1186/s40064-015-0991-x

**Published:** 2015-04-30

**Authors:** Bhupendra Koul, Reena Yadav, Indraneel Sanyal, Devindra Vijay Amla

**Affiliations:** Department of Biotechnology and Biosciences, Lovely Professional University (LPU), Jalandhar-Delhi G.T. Road (NH-1), Phagwara, 144411 Punjab India; Plant Transgenic Lab, CSIR-National Botanical Research Institute, Rana Pratap Marg, P.O. Box 436, Lucknow, 226 001 UP India

**Keywords:** *Agrobacterium tumefaciens*, Cry1Ac toxin, Tomato transformation, Leaf-disc, *Helicoverpa armigera*, Insect mortality

## Abstract

**Background:**

Bt-*cry1Ac* gene has been reputedly effective against *Helicoverpa armigera* a notorious lepidopteran pest. Reports on the expression of full-length and truncated *cry1Ac* genes in plants for effective resistance against *Helicoverpa* sp. have been documented however, their performance is still ambiguous. Moreover, the question remains to be addressed that truncation of 3′ end of the native gene was documented and suggested for active insecticidal toxin production while the most successful transgenic event(s) of commercialized-cotton are based on full-length of the *cry* gene. Therefore, we performed a comparative study on the efficacy of the two versions of *cry1Ac* genes (full-length: 3,510 bp and truncated: 1,845 bp) in T_0_ and T_1_ transgenic tomato plants and analyzed the extent of protection against *H. armigera* and also compared the results with our previous findings related to a successful transgenic tomato line Ab25E, expressing *cry1Ab* gene. The integration of *cry1Ac* gene(s) in T_0_ transgenic plants and its inheritance in T_1_ progeny was observed by PCR, RT-PCR and Southern blot hybridization analysis while, the toxin integrity, expression and toxicity was monitored by Western immunoassay, DAS-ELISA and insect bioassay respectively.

**Results:**

An average transformation frequency and Bt-Cry protein content of 16.93 ± 2.10 and 0.0020–0.0128% of total soluble protein (TSP) was obtained with pRD400 vector (Tr*cry1Ac*) while, a much lower value of 9.30 ± 2.041 and 0.0001 ― 0.0026% of TSP was observed with pNBRI-1 vector (Fl*cry1Ac*), respectively. The promising Tr*cry1Ac* T_0_ transgenic plants and their T_1_ progeny gave full protection from *H. armigera.* Although Flcry1Ac gene showed lower transformation frequency and lower expression, it showed higher toxicity to *H. armigera* when compared with truncated Tr*cry1Ac* gene.

**Conclusions:**

The full-length *cry1Ac* gene can be redesigned for higher expression and performance in dicots or a hybrid gene could be designed having a blend of strong receptor binding and stable expression characteristics for enhanced efficacy and toxicity to the susceptible insects.

**Electronic supplementary material:**

The online version of this article (doi:10.1186/s40064-015-0991-x) contains supplementary material, which is available to authorized users.

## Background

By the year 2050, the global population is expected to rise above nine billion. While, at the same time it is sure that the existing arable land is expected to decrease significantly due to anthropogenic activities related to urbanization and neglecting agricultural crop losses due to insect pests. The worldwide pre-harvest crop losses have been estimated to be 13.8% from insects and other arthropods, 11.6% from disease and 9.5% from weeds (Chrispeels and Sadava 1994). The conventional methods of crop protection rely mainly on the use of synthetic agrochemicals but, they have a significant drawback of environmental contamination and toxicity to non-target organisms, including humans themselves. It is the moral duty of environmentalists and molecular biologists to judiciously introduce healthier strategies to cope with the problem of insect pests and resistance management, for sustainable agricultural productivity.

The most widely used and well documented approach in this context is the insecticidal crystal protein (*cry*) genes of *Bacillus thuringiensis* (Bt) coding different insecticidal *δ*-endotoxins specific to different group of insects (Schnepf et al. 1998). These toxins are highly specific to the target insects, non-toxic to animals and human beings, non-hazardous and eco-friendly (Schnepf et al. 1998; Gatehouse 2008). Therefore, these are potent “biopesticides” (Sharma 2010; James 2012). The Cry1A group of toxin(s) are effective against lepidopteran insects which are the major group infecting several agricultural crop plants in field. The mode of action of Cry1Ac toxin can be best explained by the ‘Jurat-Fuentes model’ which suggests that cytotoxicity is due to the synergistic effect of osmotic lysis and cell signaling process, and involves the features of both the ‘Bravo model’ and the ‘Zhang model’ of toxin mode of action (Jurat-Fuentes and Adang 2006; Pardo Lopez et al. 2013).

Cotton transgenics developed by the transfer of modified Bt-*cry1Ac* and Bt-*cry1Ac* + *2Ab* genes and commercialized as Bollgard I and II respectively, are the great success stories in agricultural biotechnology, for providing protection against lepidopteran insects, thereby increasing the crop yield (Perlak et al. 2001; Purcell et al. 2004; Sanahuja et al. 2011). The major limitation in the development of transgenic plants with Bt-*cry1A* genes is the low expression of native gene in plants, which has been attributed to instability of transcript, poor stability of the toxin protein in plant environment and altered codon usage in plants (Murray et al. 1989). However, it is not possible to use the complete toxin encoding genes in plants because protoxins are not sufficiently soluble in plant cells due to low pH of 7.6, since higher pH above 9.5 is required for solubility of the protoxins (Peferoen 1997; Shrivastava 2012). This problem is circumvented by using *cry* genes with 3′ truncation which produce fully activated toxin molecules and remain in solubilized form in plant cell. Two approaches have been tried to increase the expression of Cry1A toxins in genetically modified plants, (1) selective removal of deleterious DNA sequences by site-directed mutagenesis and (2) completely modified sequences of *cry* genes with plant-optimized codon usage for enhanced expression in plants. The later approach has led to 100 fold increase in the expression levels of *cry1Ab* and *cry1Ac* genes in tobacco, tomato and other crop plants (Perlak et al. 1991; Koziel et al. 1993). The modified *cry* gene had about 65% nucleotide homology with the native gene while G + C content was increased from 38% to 65% along with codon optimization for over expression in higher plants. Perlak et al. (1990) had reported that the truncated *cry* gene of *B. thuringiensis* var. *kurstaki* HD-73 strain showed detectable expression compared to full-length *cry* gene in transgenic cotton (Perlak et al. 1990). The transgenic cotton plants harbouring truncated *B. thuringiensis cry1Ac* gene showed high level of activity against *Manduca sexta* resulting in 100% mortality. The laboratory trials were further confirmed and demonstrated with field trials. However, the same group reported that full-length gene rather than a truncated gene provides reasonable protection from *Heliothis virescens* (tobacco budworm) (Perlak et al. 2001; Rawat et al. 2011). They came up with an event that was used to develop Bt-transgenics resistant to damage by *Helicoverpa armigera*, the major target pest in India, China and Australia.

Reports on the expression of both the full-length and truncated *cry1Ac* genes for effective resistance against insects have been documented but, unfortunately the performance of Cry1Ac toxin encoded by full-length and truncated *cry1Ac* gene in plants is still not clear (Kranthi et al. 2005; Rawat et al. 2011). The routine recovery of transgenic events expressing high-level of Cry1Ac toxin is a rare, random and vexatious issue (Rawat et al. 2011). Also, the question remains to be addressed that truncation of 3′ end of gene was documented and suggested for active insecticidal toxin production while the most successful transgenic event(s) of cotton for field performance and commercialization are based on full-length of the *cry* gene. Therefore, we performed a comparative study on toxicity and performance of two versions of *cry1Ac* gene using tomato as a model system, for development of *H. armigera* resistant, stable transgenic plants.

## Results

### Development of transgenic tomato plants

**Figure 1:**
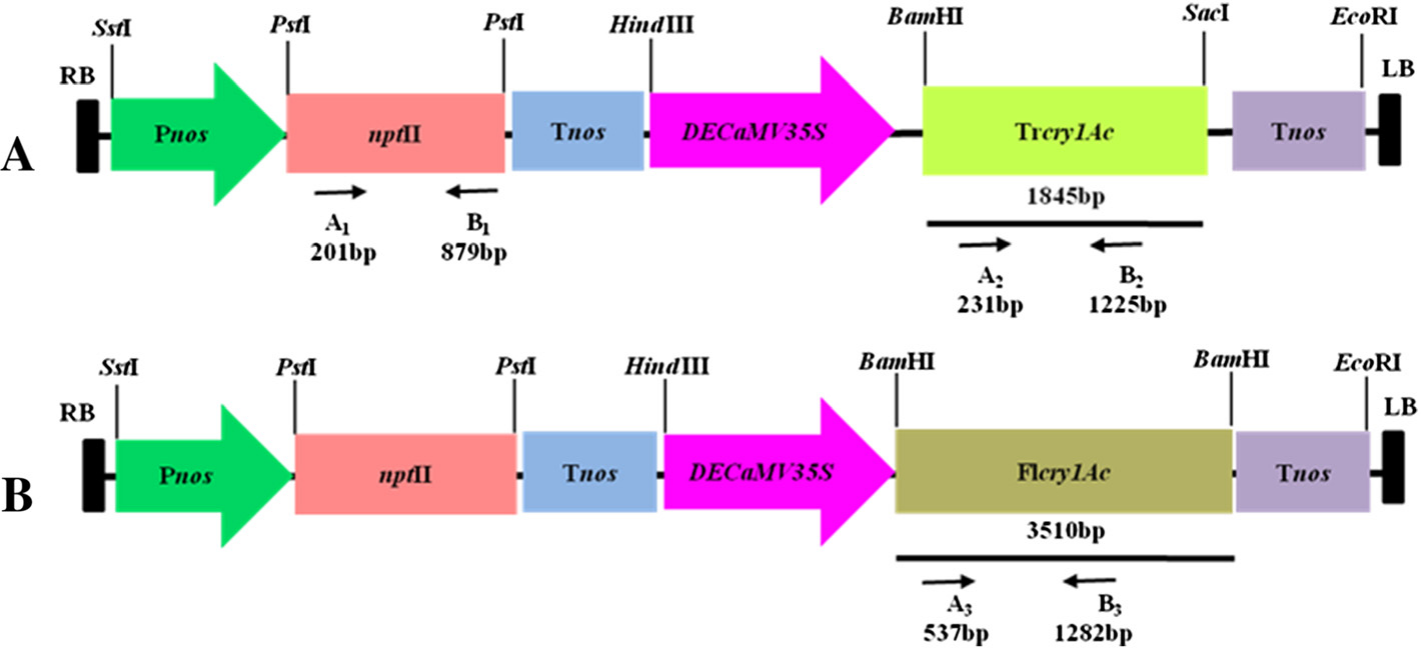
Schematic diagram of T-DNA regions of expression vectors used for tomato transformation. **A** pRD400. **B** pNBRI–1. RB and LB-right and left border sequences; *npt*II-coding region of neomycin phosphotransferase gene; *DECaMV35S*-*CaMV35S* promoter with double enhancer; *cry1Ac*-sequence coding for *cry1Ac* gene; P*nos*-promoter sequence of nopaline synthase; T*nos*-terminator sequence of nopaline synthase. Bold lines () show fragments used for DNA probe and arrows () indicates oligo primer sites used for PCR amplification denoted as A_1_ B_1,_ A_2_ B_2_ and A_3_ B_3_ respectively.

*Agrobacterium*-mediated tomato transformation was performed with vector constructs (Figure 1A and B) pRD400 harbouring truncated *cry1Ac* gene (Tr*cry1Ac*) and pNBRI–1 harbouring full-length *cry1Ac* gene (Fl*cry1Ac*) using the modified procedure. The explants which regenerated during kanamycin selection cycle (responding explants) were putative transformants. The percentage transformation frequency was determined as independent transgenic events received after the second selection divided by total number of explants, multiplied by 100. An average transformation frequency of 16.93 ± 2.10 and 9.30 ± 2.04 was observed with pRD400 and pNBRI-1 respectively, as shown in Additional file 1: Table S1. The results of molecular characterization of T_0_ and T_1_ transgenic tomato plants of the respective constructs have been discussed consecutively under respective heading: [A] Truncated *cry1Ac* gene (vector pRD400) and [B] Full-length *cry1Ac* gene (vector pNBRI–1). To avoid confusion, the transgenic events obtained with Tr*cry1Ac* were designated as Ac 1–Ac 30 and that with Fl*cry1Ac* as FlAc 1–FlAc 30.

The T_0_ transgenic plants developed with pRD400 vector harbouring Tr*cry1Ac* gene showed normal growth and flowering (43 ± 1.83 days), except the fruit size. The size of fruits was reduced compared to the control and the average weight of the fruits per plant was calculated to be 18.64 ± 0.98 gm. It was interesting to note that although the seed count was reduced with 35.4 ± 7.5 seeds (1.9 seeds per gram of tomato pulp) compared to the control tomato bearing 62.6 ± 12.60 seeds per tomato (2.57 seeds per gram of tomato pulp), but the shape and size of the seeds were normal as the control. Seed germination was not affected by the *cry1Ac* gene and the T_1_ plants attained an average height of 109.54 ± 20.80 cm with 43.20 ± 4.08 average number of leaves and 5.0 ± 2.10 average number of fruits per plant (Table 1). The T_0_ plants developed with vector pNBRI–1 harbouring Fl*cry1Ac* gene did not show any abnormality in their growth, and flowering. These transgenic plants attained an average height of 116.4 ± 6.10 cm with 56.31 ± 11.52 average number of leaves (Table 1). The size of the fruits was markedly reduced compared to the control untransformed tomato plants while the fruit number was found to be similar to that of control. The average number of fruits per plant was observed to be 16 ± 7.5 and the average weight of tomatoes was found to be 18.5 ± 1.34 gm with 29 ± 2.82 average numbers of seeds per tomato (1.56 seeds per gram of tomato pulp).

**Table 1:** **Comparative assessment of growth parameters of T**_**0**_ **plants developed with vectors pRD400 and pNBRI–1**

**Vector construct**	**Cry toxin (% of tsp)**	**Average plant height**	**Average number of leaves**	^**a**^ **Onset of flowering (days after hardening)**	^**b**^ **Average number of fruits/plant**	**Number of seeds/fruit**	^**c**^ **Dry weight yield (gm)**	^**d**^ **Number of seeds/gm of fruit**	**% germination of T**_**1**_ **seeds**
Control	–	106.4 ± 6.87	42.00 ± 7.21	30 ± 1	14.0 ± 1.93	62.6 ± 12.60	520 ± 8.832	2.57	75 ± 2.95
pRD400	0.0020–0.013	109.54 ± 20.8 (0.373)	43.20 ± 4.08 (0.3767)	43 ± 1.83 (0.000054)	5.0 ± 2.10 (0.000014)	35.4 ± 7.5 (0.00257)	470 ± 5.47 (0.00204)	1.9	49 ± 2.58
pNBRI–1	0.0001–0.003	116.40 ± 6.10 (0.0205)	56.31 ± 11.52 (0.010)	36 ± 1.41 (0.0079)	16.0 ± 7.5 (0.2256)	29.0 ± 2.32 (0.000006)	496 ± 10.95 (0.0047)	1.57	32 ± 3.00

^a^Overall average of the days of onset of flowering, in 30 transgenic tomato plants of each group.

^b^Average number of fruits per plant in 30 transgenic tomato plants of each group.

^c^Average dry weight of 30 transgenic tomato plants of each group of plants.

^d^Average number of seeds (per gram of fruit) in eight fruits of individual transgenic plant, in 30 plants of each group.

Values in parenthesis indicate the probability associated with a student’s paired t-test with a two tailed distribution. When P < 0.05 the difference of the individual parametric value to that of the value of the control was significant.

### [A] Truncated *cry1Ac* gene (vector pRD400)

#### Molecular characterization of transformants

**Figure 2:**
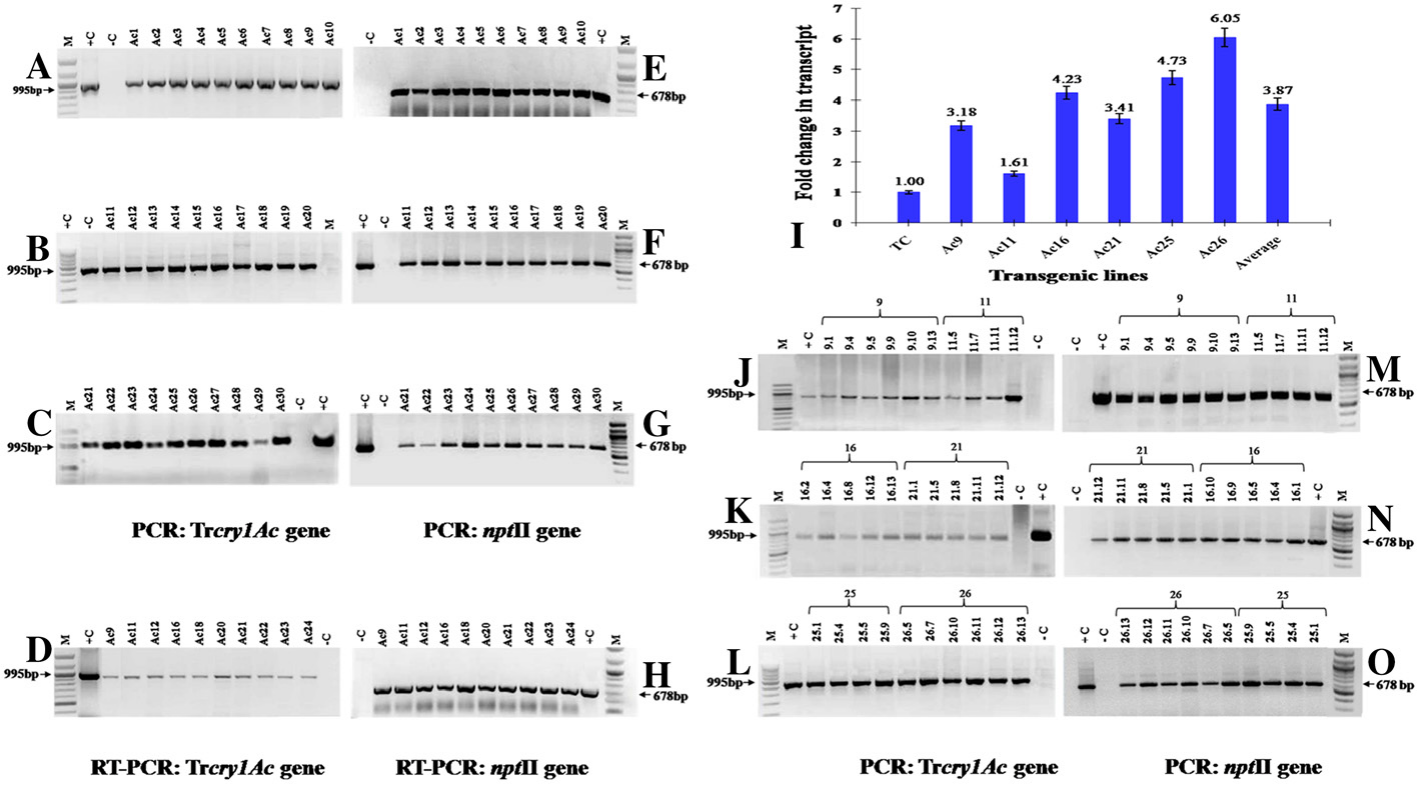
Screening of T_0_ and T_1_ Tr*cry1Ac* transgenic tomato plants. **A–C** PCR amplification of 995 bp of *cry1Ac* gene. **D–F** 678 bp of *npt*II gene using gene specific primers in thirty T_0_ transgenics. **G**, **H** RT-PCR analysis of ten randomly selected T_0_ transgenic tomato plants of *cry1Ac* showing 995 bp *cry1Ac* gene and 678 bp *npt*II gene transcripts. M – 100 bp DNA ladder (NEB, USA). -C – non-transgenic control plant, +C – plasmid DNA positive control. **I** Real-time analysis for Bt-*cry1Ac* transcript levels in six T_0_ transgenic tomato plants. TC – Transgenic plant with low expression of Bt-toxin used as control. **J–O** PCR amplification of 995 bp of *cry1Ac* gene and 678 bp of *npt*II gene using gene specific primers inT_1_ progeny of promising T_0_ parents. M −100 bp DNA ladder (NEB, USA). –C : non-transgenic control plant, +C : plasmid DNA positive control.

A total of thirty independently selected T_0_ transgenic tomato plants were screened for the presence of *cry1Ac* and *npt*II gene by PCR amplification using the specific set of primers. These plants showed expected amplicon of 995 and 678 bp for *cry1Ac* and *npt*II genes respectively (Figure 2A–C,E–G). The presence of both the genes (*cry1Ac* and *npt*II) indicated intact integration of the transgene and the selection marker. Out of thirty T_0_ transformants RT-PCR analysis of ten randomly selected plants was performed to confirm the formation of *cry1Ac* and *npt*II gene transcript. The first strand cDNA was amplified using specific set of primers which showed expected amplicons of 995 and 678 bp for *cry1Ac* and *npt*II genes respectively (Figure 2D,H).

### Determination of transcript level by real-time PCR

The transcript level was analyzed by real time PCR in seven randomly selected T_0_ transgenic tomato plants expressing *cry1Ac* gene. The expression level of plant ID Ac1 was very low and was therefore taken as a reference which was denoted as TC (transformed control). The comparative transcript level ranged from 2.18 (Ac 9), 0.61 (Ac 11), 3.2 (Ac 16), 2.4 (Ac 21), 3.7 (Ac 25) and 5.1 (Ac 26) folds higher to Ac 1 respectively (Figure 2I). No amplification was observed in non-transformed control plant and on an average the transgenic lines showed 2.9 folds enhanced expression over the reference (Ac 1).

### Southern blot hybridization analysis and Western immunoassay

**Figure 3:**
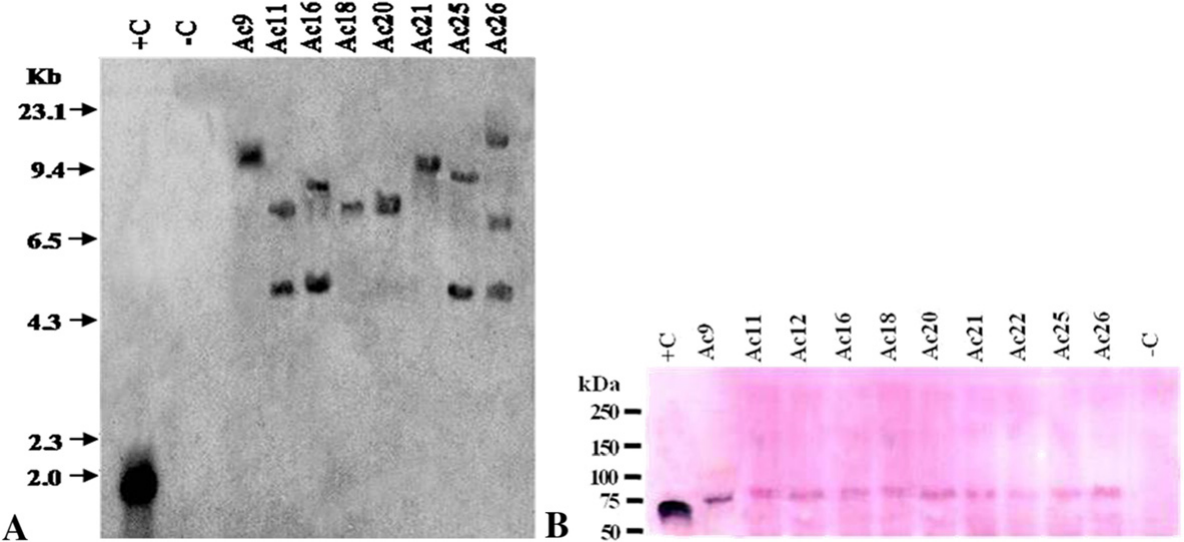
Southern blot hybridisation and Western immunoblot analysis of T_0_ Tr*cry1Ac* transgenics. **A** Southern blot hybridisation analysis of eight T_0_ transgenic tomato plants of *cry1Ac* probed with 1,845 bp radiolabelled *Bam*HI-*Eco*RI fragment of *cry1Ac* gene. +C 1,845 bp *cry1Ac* gene fragment; −C Untransformed control plant. **B** Western immunoblot analysis of ten T_0_ transgenic plants, with crude protein extract; lane 1: purified Cry1Ac protein; lane 12 : untransformed control plant protein; lane 2–11: protein samples of ten transgenic plants. The transgenic samples gave a band of ~65 kDa, similar to the positive control. –C : Untransformed control plant.

The transformants confirmed by PCR and RT-PCR were further analyzed by Southern blot hybridization. The genomic DNA from transformed and untransformed plants was digested with *Eco*RI as there is a unique site for this restriction enzyme in the T-DNA region of the binary vector pRD400. Southern blot hybridization of eight T_0_ plants with 1,845 bp *Bam*HI and *Eco*RI fragment of *cry1Ac* gene probe revealed that they were independent transgenic events. Most of the promising T_0_ transgenic plants showed single and double copy insertion while only one plant (Ac 26) showed triple copy insertion of the transgene and the hybridizing fragments ranged from 4.5–15.5 kb (Figure 3A). Whereas, genomic DNA from untransformed control tomato plants did not show any hybridization signal with *cry1Ac* gene probe. Western immunoassay of ten transgenic plants including those 8 plants which passed the Southern blot hybridization analysis was performed using the cell-free extract of leaf tissues. The protein blots showed a light band of approximately 65 kDa which was similar to the positive control (Figure 3B).

### Protein expression and corresponding insect mortality

**Figure 4:**
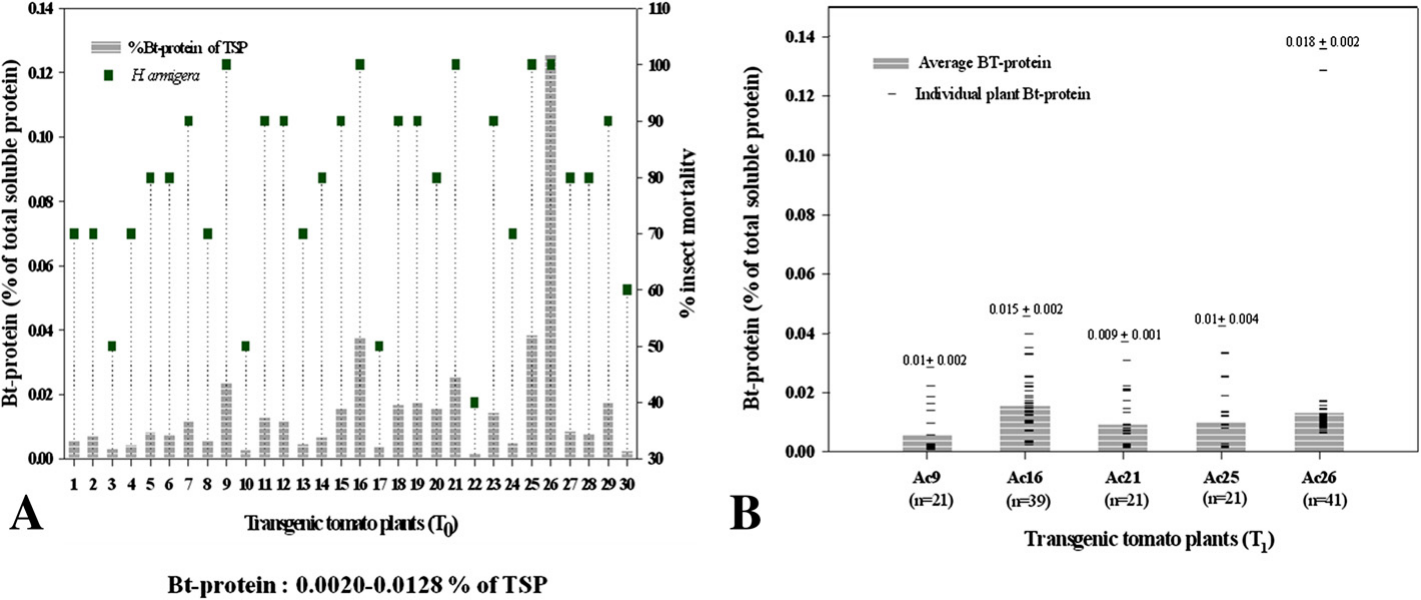
Expression of Tr*cry1Ac* gene in transgenic plants. **A** Thirty T_0._ **B** Selected T_1_ transgenics. The gray shaded vertical bar represents the average content of Bt-Cry1Ac protein. The squares (green square) represent the average of the % mortality status of *H. armigera*, when subjected to the leaves of T_0_ and respective T_1_ transgenic tomato plants. ‘n’ is the number of T_1_ transgenic population from each parent.

The expression level of *cry1Ac* gene in T_0_ and T_1_ transgenic tomato plants was analyzed through DAS-ELISA and calculated as percentage of Bt-protein of total soluble protein (TSP). Cry1Ac toxin content in thirty independent T_0_ plants ranged between 0.0020–0.0128% of TSP. The resistance bestowed against fruit worm *H. armigera* corresponding to the Cry1Ac toxin is shown in Additional file 2: Table S2 and Figure 4A.

Few T_0_ plants expressing Cry1Ac toxin above 0.02% of total soluble protein (TSP) with plant ID Ac 9 (0.0240%), Ac 16 (0.0380%), Ac 21 (0.0255%), Ac 25 (0.0386%) and Ac 26 (0.1259%), were selected for further analysis of their T_1_ progeny. The T_1_ seeds of these transgenics plants (plant ID Ac 9, Ac 11, Ac 16, Ac 21, Ac 25 and Ac 26) were grown on kanamycin selection media to study the segregation pattern and further molecular characterization of the segregated kanamycin resistant population. These highly expressing T_1_ progeny of respective T_0_ parents gave PCR amplification of the expected amplicon of 995 and 678 bp for *cry1Ac* and *npt*II genes, respectively (Figure 2J–L,M–O). The average Cry1Ac toxin content of the progeny of Ac 9, Ac 16, Ac 21, Ac 25 and Ac 26 parents showed 0.01 ± 0.002, 0.015 ± 0.002, 0.009 ± 0.001, 0.01 ± 0.004 and 0.018 ± 0.002% of TSP respectively (Figure 4B) causing 100% mortality of *H. armigera* after 72 h of feeding on detached vegetative leaves. The control non-transgenic leaves suffered heavy damage due to voracious feeding by the insect. (Figure 4A,B).

Segregation of *npt*II gene in T_1_ progeny was studied in these five highly expressing ELISA positive, independent transformants as shown in Additional file 3: Table S3. It was observed that the segregation ratio was Mendelian in nature with a minimum Chi-square value of 0.19 for Ac 25 and maximum value of 3.34 for Ac 16.

### [B] Full-length *cry1Ac* gene (vector pNBRI–1)

#### Molecular characterization of the T_0_ transgenic plants

**Figure 5:**
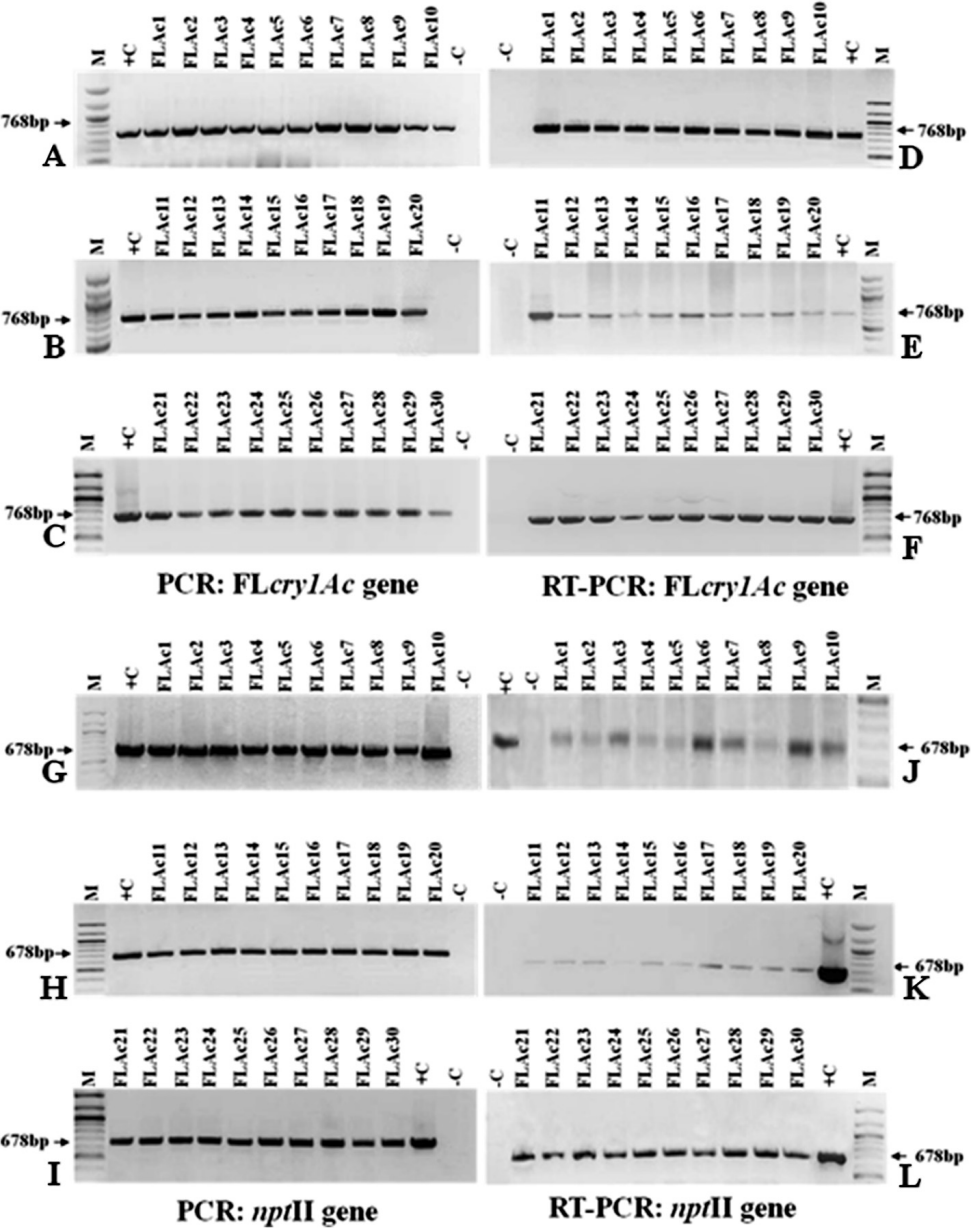
Molecular characterizations of T_0_ Fl*cry1Ac* transgenic tomato plants. *Upper Panel* **A–C** PCR amplification of 768 bp of Fl*cry1Ac* gene using specific primers in thirty T_0_ transgenics. **D–F** RT-PCR analysis of T_0_ transgenic tomato plants showing 768 bp amplicon of *cry1Ac* gene. M : 100 bp DNA ladder (NEB, USA). -C : non-transgenic control plant, +C : plasmid DNA positive control. *Lower Panel* **G–I** PCR amplification of 678 bp *npt*II gene using specific primers in T_0_ transgenics. **J–L** RT-PCR analysis of T_0_ transgenic tomato plants of showing 678 bp amplicon of *npt*II gene. M : 100 bp DNA ladder (NEB, USA). -C : non-transgenic control plant, +C : plasmid DNA positive control.

A total of thirty T_0_ transgenic plants were taken for the population study and were screened for the presence of *cry1Ac* gene through PCR. All the plants were PCR positive and showed an expected amplicon of 768 bp gene specific primers (Figure 5A–C). RT-PCR analysis of these plants was performed and the cDNA was amplified using gene specific primers, showing an expected amplicon of 768 bp (Figure 5D–F). These T_0_ plants were also screened for the presence of *npt*II gene and PCR amplification gave an expected amplicon of 678 bp (Figure 5G–I). Similarly, RT-PCR analysis for the *npt*II gene transcript also showed 678 bp amplicon of *npt*II gene (Figure 5J–L).

### Protein expression and corresponding insect mortality

**Figure 6:**
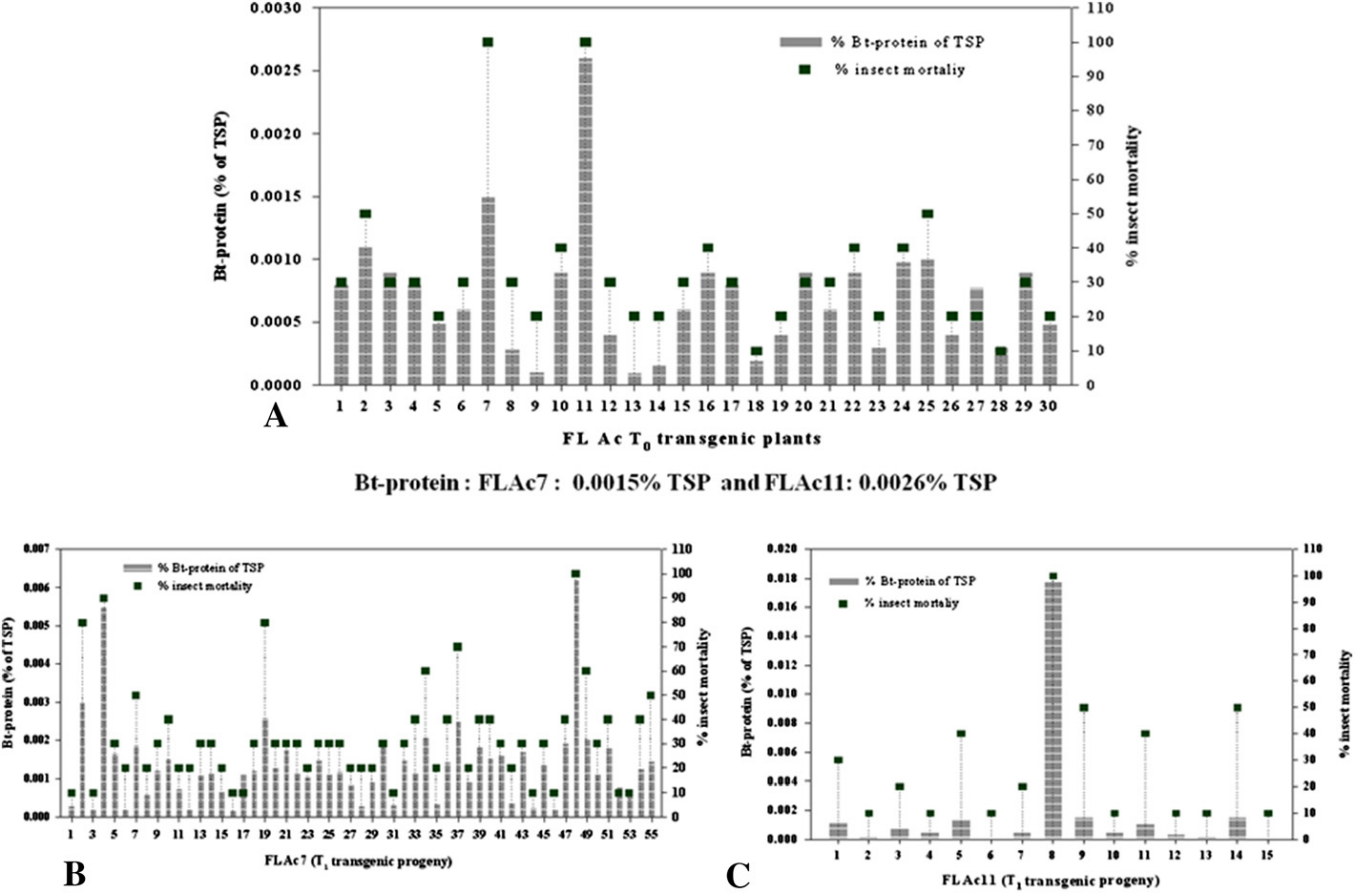
Expression of Fl*cry1Ac* gene in transgenic plants. **A** Thirty T_0_ transgenics. **B**, **C** T_1_ progeny of FLAc 7 and 11. The gray shaded vertical bars represents the average content of Bt-Cry1Ac protein and squares (green square) represent the average of the % mortality status of *H. armigera*, when subjected to the leaves of T_0_ transgenic plants.

Bt-ELISA of all the PCR and RT-PCR positive thirty T_0_ transgenics was performed using pathoscreen cry1Ab/AC ELISA kit (Agdia, USA). Interestingly, only two plants, FLAc 7 and FLAc 11 showed detectable expression of Bt-protein content which was estimated to be 0.0015 and 0.0026% of total soluble protein (TSP), respectively (Figure 6A). Detached leaf bioassay of FLAc 7 and FLAc 11 was performed with second instar larvae of *Helicoverpa armigera*. Both these plants gave 100% mortality to the larvae in repeated bioassays (Additional file 2: Table S2 and Figure 6A). The leaves of FLAc 7 and FLAc 11 were also subjected to second instar larvae of *Spodoptera litura* and caused 20% and 30% insect mortality to the larvae, respectively (data not shown). This result indicates the specificity of *cry1Ac* gene for *H. armigera* midgut receptors. These two plants FLAc 7 and 11 were selected for further analysis and segregation pattern of the kanamycin selection marker. Segregation of *npt*II gene in T_1_ seeds of the two ELISA positive independent transformants FLAc 7 and FLAc 11, was analysed, as shown in Additional file 3: Table S3. The segregation ratio was Mendelian in nature with a minimum Chi-square value of 0.82 for FLAc 11 and maximum value of 1.25 for FLAc 7.

Fiftyfive T_1_ plants of FLAc 7 and fifteen plants of FLAc 11 were screened through DAS-ELISA. As expected, all the T_1_ transgenic tomato plants were found to be ELISA positive. The Bt-protein content of few highly expressing plants FLAc 7.2, 7.4, 7.19, 7.34, 7.37, 7.48, 11.8 , 11.9, 11.11 and 11.14 was 0.003%, 0.0055%, 0.0026%, 0.0021%, 0.0025%, 0.0062%, 0.0178%, 0.0015%, 0.001 and 0.0015% of TSP, respectively (Figure 6B,C). These plants were also subjected to insect bioassay with *H. armigera* and varying degrees of protection corresponding to the expression level of Bt-protein was observed. Only FLAc 7.48 and FLAc 11.8 gave 100% mortality to second instar larvae of *H. armigera* while FLAc 7.2, FLAc 7.4, FLAc 7.19, FLAc 7.34, FLAc 7.37 and FLAc 11.4 bestowed 80%, 90%, 80%, 60%, 70% and 50% protection respectively, against the insect (Figure 6B,C).

### Determination of transcript level by real-time PCR

**Figure 7:**
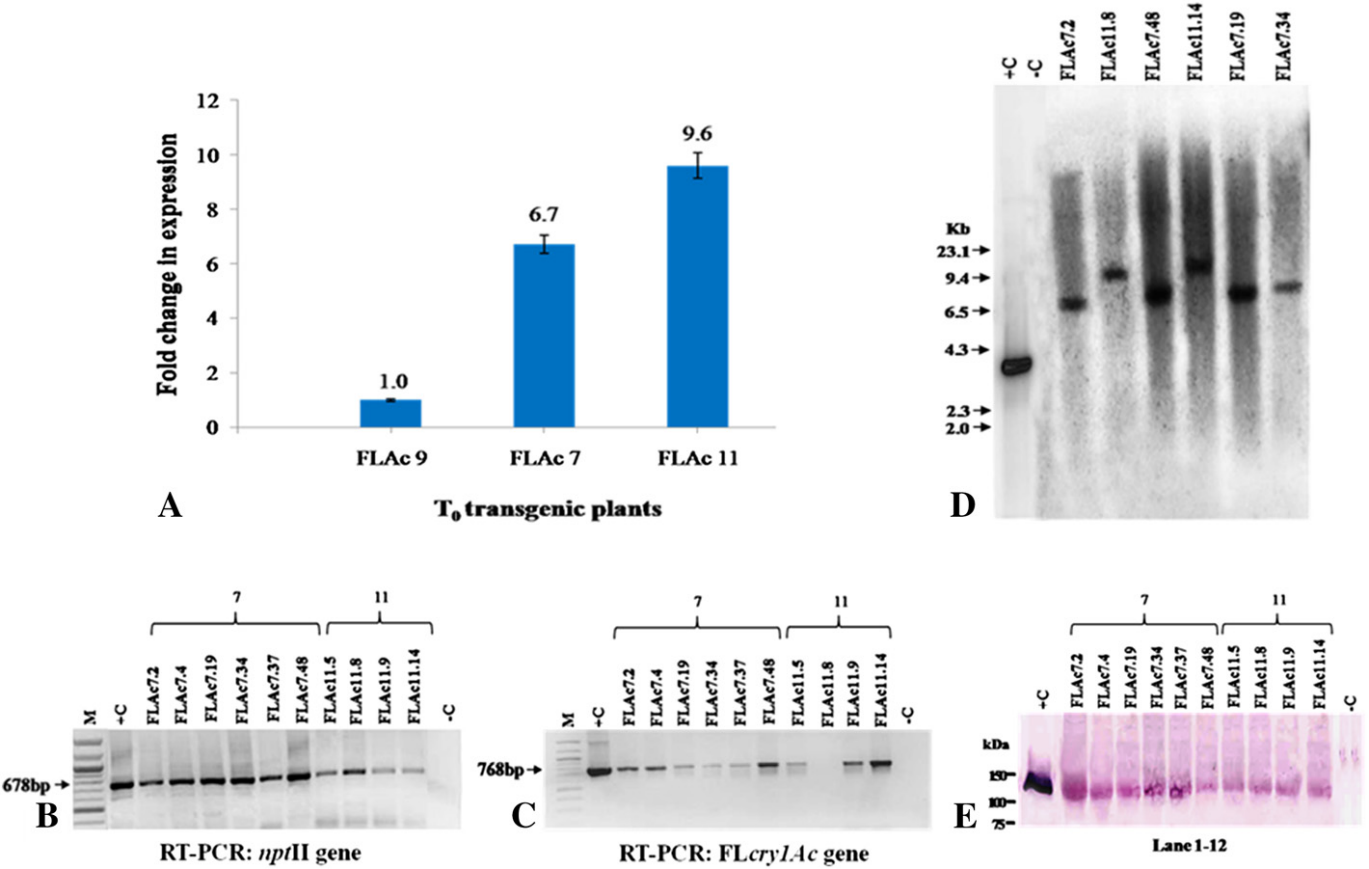
Molecular characterizations of T_0_ and T_1_ progeny of FlAc7 and FlAc11 transgenic tomato plants. **A** Comparative real-time PCR analysis of transcript in T_0_ Fl*cry1Ac* transgenic plants showing fold change in expression with respect to FlAc 9 (low expressing transgenic plant taken as reference). Control : non-transgenic control. **B**, **C** RT-PCR and cDNA amplification of 678 bp *npt*II gene and 768 bp Fl*cry1Ac* gene of T_1_ progeny using specific primers. M : 100 bp DNA ladder (NEB, USA). -C : non-transgenic control plant, +C : Fl*cry1Ac* gene plasmid DNA as positive control. **D** Southern blot probed with 3,510 bp *Bam*HI radiolabelled frament of FlCry1Ac gene. **E** Western immunoblot assay performed with crude leaf protein extract, lane1 purified Cry1Ac toxin protein, lane 12 : untransformed control, lane 2–11 : leaf protein extracts from progeny of T_0_ FlAc7 and FlAc11. A protein band of ~130 kDa in transgenic plants showed hybridization with Cry1Ac antibodies, similar to positive control.

The two T_0_ transgenic tomato plants, FLAc 7 and FLAc 11 were also subjected to quantitative real-time PCR. The expression level of T_0_ transgenic tomato plant ID FLAc 9 was very low and was therefore taken as a reference which was denoted as TC (transformed control). The comparative transcript level in transgenic plants ranged from 6.7 (FLAc 7) and 9.6 (FLAc 11), folds higher to FLAc 9 respectively (Figure 7A). No amplification was observed in non-transformed control plant and on an average the transgenic lines showed 8.1 folds enhanced expression over the reference (FLAc 9).

### Molecular characterization of the T_1_ progeny of promising T_0_ transgenic plants

All the ELISA positive T_1_ plants FLAc 7.2, FLAc 7.4, FLAc 7.19, FLAc 7.34, FLAc 7.37, FLAc 7.48, FLAc 11.8 and FLAc 11.14, expressing Bt-Cry1Ac toxin above 0.003%, were subjected to RT-PCR analysis using gene specific primers. All the plants showed expected amplicon of 678 bp for *npt*II gene and 768 bp for Fl*cry1Ac* gene (Figure 7B,C). Southern blot hybridization was performed with six T_1_ plants (FLAC 7.2, FLAc 11.8, FLAc 7.48, FLAc 11.14, FLAc 7.19 and FLAc 7.37) using 3,510 bp Fl*cry1Ac* gene fragment as probe. The genomic DNA from transformed and untransformed plants was digested with *Sal* I as there is a unique site for this enzyme in the T-DNA regions of the binary vector pNBRI–1. The hybridizing fragments ranged between 6.5–10.0 kb and showed single copy integration of the transgene (Figure 7D). Western immunoblot analysis of T_1_ progeny of FlAc 7 and FlAc 11 revealed a specific but fuzzy band of approximately 130 kDa, in all the plant samples. The non-transgenic control lane did not show any colour signal (Figure 7E). The T_1_ transgenic plant FLAc 11.8 showed complete protection against second instar larvae of *Helicoverpa armigera* and was finally selected as a promising event (Figure 6C).

## Discussion

The first transgenic crop that was commercialized in the USA was Bt-cotton. It was developed to express full-length synthetic *cry1Ac*-like *Bt*-gene sequence and its worldwide acceptance has grown exponentially since its introduction. This product has significantly reduced cotton production costs and the recurrent use of pesticides by providing a promising alternative for the control of *Heliothis virescens*, *Helicoverpa zea*, and *Pectinophora gossypiella*. Bt-cotton ensures considerable agronomic, economic and environmental benefits to its growers. The information on Cry-toxin receptors, the stability and efficacy of different Cry-toxins and the resistance mechanisms developed by the target pests is crucial to maintain the utility of *Bt-*transgenic technology.

There are few reports available in order to prove the efficacy and the stability of native full-length *cry1Ac* gene and its expression, in transgenic plants (Barton et al. 1987; Vaeck et al. 1987; Perlak et al. 1990; De Rocher et al. 1998). In most of the reports the research groups have tried to depict the performance of the *Bt*-genes in T_0_ primary transformants or did not perform a population study in subsequent progenies to confirm the stability of *cry* gene expression in terms of growth characteristics and inheritance. Our results obtained from PCR, Southern blotting and RT-PCR analyses of transgenic tomato plants have confirmed the stable integration of Tr*cry1Ac* and Fl*cry1Ac* genes in T_0_ plants and their respective T_1_ progeny. The different sizes of hybridizing genomic DNA fragments (~4.5–15.5 kb) of transgenic tomato plants with the respective *cry* gene-probe indicated that they resulted from the independent stable T-DNA integration event into the plant genome. The Bt-toxin content of T_0_ transgenic population of tomato developed with modified Tr*cry1Ac* and modified Fl*cry1Ac* genes ranged between 0.0024–0.126%, and 0.0001–0.0026% of TSP, respectively. Although, all the T_0_ transgenics were PCR and RT-PCR positive however, a remarkable difference was observed in the number of true transgenics (transgenics with detectable expression) obtained with equal number of transformation experiments and overall transformation frequencies obtained with both vector constructs. A higher transformation frequency of 16.93% ± 2.10 was observed with vector harbouring modified Tr*cry1Ac* gene and a much lower value of 9.30 ± 2.041 with vector harbouring Fl*cry1Ac* gene. The level of transgene expression in plants is generally unpredictable and may vary with different vector-constructs and also independent transformants with same vector-construct.

The T_1_ seeds of Tr*cry1Ac* transgenic tomato were subjected to kanaycin selection. It was observed that the average Cry1Ac toxin content of the progeny of Ac 9, Ac 16, Ac 21, Ac 25 and Ac 26 were less than their respective parents. After segregation of the Bt-transgene very few candidate plants retained expression level similar to their respective parents. There are certain factors which do influence transgene expression and its stability in transgenic plants and may lead to highly variable expression within populations of plants from individual parents, developed in the same transformation experiment. The position effect being the most important factor reflects the significant role of genomic DNA adjoining the site of transgene integration (Wilson et al. 1990). Another factor is the copy number, intactness of the transgenes and their relative arrangement, which influences the probability of physical interactions, recombination within the locus and the induction of gene silencing (Hobbs et al. 1993; Heinrichs 2008). The probable cause for low expression of the Tr*cry1Ac*-T_1_ population is, during segregation the transgenic locus is directed to or near a heterochromatin rich region thereby producing variants with a lower and varied Bt-content. But in case of Fl*cry1Ac* transgenics, the gene shows single copy integration and is segregated along the gene rich euchromatin region of the chromosome.

Our results also suggest that Fl*cry1Ac* gene is comparatively poorly expressed than Tr*cry1Ac*, despite the optimal conditions of transformation and the overall transformation frequency was low. An interesting observation was noticed when T_0_ transgenics developed with pRD400 and pNBRI–1 vector-constructs were subjected to insect bioassay with *H. armigera*. Although highly expressing transgenic plants expressing Tr*cry1Ac* gene showed protection against *H. armigera* whereas, only two low expressing transgenic events harbouring Fl*cry1Ac* gene, (FlAc7 and FlAc11) repeatedly showed full-protection against *H. armigera*. This observation made us to realize that although Fl*cry1Ac* gene showed a lower transformation frequency and expression but the promising events were highly toxic to *H. armigera*. Certainly, there is a scope for more improvement of Fl*cry1Ac* gene for higher expression in transgenic plants. These results can be co-related to the most successful transgenic event Monsanto 531 which was developed with full-length modified *cry1Ac*-like gene and commercialized as Bollgard cotton, for complete protection against bollworm complex (Perlak et al. 2001; Purcell et al. 2004).

There are not many reports concerning the development of promising transgenic plants over-expressing variants of Cry1Ac toxin (native truncated and native full-length or modified and truncated or modified full-length). It may be attributed to toxic or suppressive response of Cry1Ac toxin during early stage of plant cell development (Barton et al. 1987; De Rocher et al. 1998; Rawat et al. 2011). It was reported by Rawat et al. 2011 that high expression of *cry1Ac* gene acts as a negative selection and regenerated plants cells with low expression of the gene are the ones which actually proliferate. The same group reported that certain morphological abnormalities occur in *in vitro* regeneration of explants after transformation with *cry1Ac* gene and in the growth of the transgenic plants. Our results of transformation frequency with Fl*cry1Ac* gene can be compared with it. In the present study, although the transformation frequency was reduced but we did not come across morphological abnormalities in the regenerating explants transformed with *cry1Ac* genes. The only growth related abnormality which was observed in few T_0_ plants during glass house-stage was their delayed flowering and fruit setting. This problem was confirmed after observing the floral morphology of pRD400 transformants where the stigmal apparatus was longer than the filaments and projected outside the unopened flower. As tomato is strictly self pollinated crop because of cleistogamous nature of flowers, the stigma and style should reside within the unopened flower. But, to our surprise the T_1_ generation did not face the same problem. In the study of Sachs et al. (1998) involving the inheritance of the *cry1Ac* gene in MON249 event in cotton, it has been hypothesized that reduced fitness of some of the transgenic lines may be a result of direct insertion effects leading to the down-regulation of one or more native genes or the result of a linked somaclonal variation (Sachs et al. 1998).

It was interesting to note that apart from a low Bt-toxin protein content Fl*cry1Ac*-transgenics gave full protection to *H. armigera* which was comparable to Tr*cry1Ac*-transgenics expressing higher levels of Bt-toxin. In a recent study of Gomez et al. (2014) on *Manduca sexta* insect bioassay, they have reported that the insecticidal activity of modified Cry1Ab toxin (active toxin) was 8-fold lower than the modified Cry1Ab protoxin. Their experimental data show protoxin molecules trigger the formation of pre-pore structures and supports the pore-formation model involving sequential interaction with different midgut receptor which culminates to pore formation in the gut membrane and insecticidal activity. These findings could be related to our study and the question as to why the full-length Cry1Ac toxin at a lower concentration is effective than the truncated Cry1Ac toxin can be answered. The modified full-length Cry1Ac toxin, although at a lower expression levels, efficiently induces oligomerization, pre-pore formation and insecticidal activity compared to modified truncated Cry1Ac toxin at higher expression levels. These results suggest the importance of modified full-length *cry1Ac* gene for stability and integrity of the insect-resistance trait compared to truncated version of *cry1Ab* or *cry1Ac* genes alone (Koul 2013). Thus, the functional role of protoxin segments in the pore formation is yet to be analysed.

The truncated *cry1Ab* gene bears >80% homology with truncated *cry1Ac* gene. In our previous findings, we raised Bt-*cry1Ab* transgenic tomatoes and performed similar tests. A maximum transformation frequency of 28.20% was obtained with binary vector pBIN200 harbouring the modified truncated 1,845 bp *cry1Ab* gene (Koul 2014b). The best transgenic line Ab25 E, expressing 0.47 ± 0.01% Cry1Ab toxin of total soluble protein (TSP) was finally selected in the T_1_ generation from the segregating population showing 100% mortality to the second instar larvae of *H. armigera* and *S. litura* and minimal damage to leaves and fruits (Additional file 4: Figure S1). The success of transgenic event Ab25E expressing modified truncated *cry1Ab* may be attributed to possible incorporation of *cry1Ab* transgene in euchromatin hot spot region of the genome. The position effect on transgene expression probably reflects pre-existing features of the insertion site, such as proximity to genome enhancers and degree of chromatin condensation (Beaujean et al. 1998). In tomato Cry1Ab25 E event the same phenomena seems to be applicable where stable integration accompanied by functional stability of the transgene made the event a successful line.

The commercially released Bt-cotton was developed with full-length *cry1Ac-*like gene whose nucleotide alignment study revealed that ‘Monsanto 531’ *cry* gene sequence is a hybrid gene where the sequence 1–1398 bp is that of *cry1Ab* gene. We can easily summarize that it was done in order to provide a blend of binding as well as pore formation characteristics in this successful *cry* gene for raising transgenic cotton and its commercialization. The Bt-*cry1Ab* gene offers higher transformation frequency (24.98 ± 3.56), optimal expression (0.02–0.13% of TSP) and good receptor-binding characteristics and is a promising candidate gene for gene pyramiding strategy to delay insect resistance.

## Conclusions

The Cry1Ac toxin has been reputedly effective against lepidopteron insects, especially *H. armigera* (Mandaokar et al. 2000). In the present study, although, the expression of Tr*cry1Ac* gene was 100 folds higher than the Fl*cry1Ac* gene the latter gave full-protection from *H. armigera* at very low concentration as evident from promising T_0_ plants (plant ID FlAc7 and FlAc11) and their T_1_ transgenic population study. The functional role of protoxin segments in Cry-pore forming toxin activity is yet to be studied in detail. The full-length *cry1Ac* gene can be redesigned for higher expression and stability in crop plants and can be pyramided with other *cry* gene(s) or a hybrid gene can be designed to broaden its toxicity spectrum and efficacy, as a remedy to cope with the problem of insect resistance.

## Methods

### *Agrobacterium* strain and gene constructs

*Agrobacterium tumefaciens* strain LBA4404 harbouring binary vector pRD400, with modified and truncated 1,845 bp *cry1Ac* gene having 47.74 % GC content (Sardana et al. 1996; courtesy provided by Prof. I. Altosaar, University of Ottawa, Ottawa, Canada) and pNBRI–1 with modified full-length 3,510 bp *cry1Ac* gene 47.89% GC content (Koul 2013) respectively, driven by double enhancer DE*CaMV35S* promoter and neomycin phosphotransferase gene (*npt*II) for kanamycin resistance in pBIN 20 backbone (Hennegan and Danna 1998) has been used for tomato transformation, as shown in Figure 1A and B. Cultures of *A. tumefaciens* were grown at 28°C in YEB medium containing 20 mg l^−1^ rifampicin, 50 mg l^−1^ kanamycin and 50 mg l^−1^ streptomycin for 24 h at 200 rpm and utilized for transformation of tomato leaf-discs.

### Plant material

Breeder seeds of *Solanum lycopersicum* cv. Pusa early dwarf (PED) were obtained from National Seeds Corporation, New Delhi, India and used for *Agrobacterium*-mediated leaf-disc transformation of tomato.

### Tomato transformation and plantlet regeneration

Tomato seeds were surface sterilized and placed on semi-solid MS medium (Murashige and Skoog 1962), containing B5 vitamins (Gamborg et al. 1968) 3% (w/v) sucrose (HiMedia Labs, Mumbai, India) and 8 g l^−1^ agar (Sigma, USA) followed by incubation at 24 ± 2°C in dark and shifted after three days of 16:8 h light–dark cycle in culture room maintained at 22 ± 2°C, illuminated with light intensity of 100 μmol m^−2^ s^−1^ and 78 ± 4% relative humidity. Vegetative leaves from axenic tomato seedlings of 16–18 days, were excised and initially pre-cultured on MS medium supplemented with 2.5 mg l^−1^ 6-benzyladenine (BAP) + 0.5 mg l^−1^ indole-3-acetic acid (IAA) for three days prior to *Agrobacterium* co-cultivation (Koul 2013).

*Agrobacterium*-mediated transformation of tomato was performed by the method described by Koul et al. 2014a, 2014b. Tomato leaf discs were dipped in *Agrobacterium* suspension OD_600_ ≈ 0.25–0.3 (2 × 10^9^ cfu ml^−1^), in MS liquid co-cultivation medium supplemented with 100 μM acetosyringone (As) for 20 min. The leaf disc explants were dried on sterilized blotting paper and transferred onto semi-solid co-cultivation medium comprising of MS salts + 3% (w/v) maltose + 100 μM As + 2.5 mg l^−1^ BAP + 0.5 mg l^−1^ IAA and co-cultivated in dark, for two days in the culture room. The explants thereafter were incubated on medium consisting of MS salts + 3% (w/v) maltose + 500 mg l^−1^ cefotaxime + 2.5 mg l^−1^ BAP + 0.5 mg l^−1^ IAA for 5–7 days and transferred to shoot induction medium one (SIM-1) containing MS salts + 3% (w/v) maltose + 2.5 mg l^−1^ BAP + 0.5 mg l^−1^ IAA + 250 mg l^−1^ cefotaxime + 50 mg l^−1^ kanamycin and incubated for 30 days for the first screening of putative transgenic plants (I^st^ selection). The independent regenerated shoots with a pair of vegetative leaves developed on first cycle of kanamycin screening were identified. These first pair of vegetative leaves were excised and sub-cultured on the shoot induction medium supplemented with kanamycin (SIM–2) having the same constituents as SIM–1 and incubated for 30 days for subsequent direct second screening and selection. The independent shoots that regenerated after successive II^nd^ selection cycles were sub-cultured on shoot elongation medium (SEM) containing MS medium + 1.0 mg l^−1^ gibberellic acid (GA_3_) + 3% (w/v) sucrose and 50 mg l^−1^ kanamycin. The transformation frequency percentage for each experiment with respective vector-construct was calculated as total putative transgenic shoots developed after second selection divided by total number of leaf disc explants used, multiplied by 100. The shoots recovered from SEM medium having 2–3 leaves were transferred to root induction medium (RIM) containing half-strength MS medium + 0.5 mg l^−1^ indole-3-butyric acid (IBA) + 50 mg l^−1^ kanamycin + 2% (w/v) sucrose and 0.8% (w/v) agar for 14 days. The rooted plantlets were transferred to plastic pots containing sterilized soilrite (Keltech Energies Ltd. Bengaluru, India) and irrigated with half-strength liquid MS medium devoid of sucrose. The pots were kept in a plant growth chamber (Conviron Adaptis 1000 PG, Canada) set at desired relative humidity starting from 90 to 70% for 14 days of hardening step and the hardened plantlets were potted in earthen pots filled with soil:sand:farmyard manure (in 3:1:1 ratio) and transferred to glasshouse maintained at 24 ± 1°C under natural light for normal development, flowering and seed setting.

The seeds of promising T_0_ transgenic plants of tomato were graded on the basis of quantitative Cry1Ac toxin expression, processed, air dried and kept under vacuum at 24 ± 2°C. The segregation and selection of promising events in subsequent generations from each transgenic plant were screened on kanamycin-supplemented semi-solid medium and Cry1Ac toxin level was estimated by double antibody sandwich enzyme-linked immunosorbent assay (DAS-ELISA). The selected promising transgenic plants showing higher expression of Cry1Ac toxin, normal growth and flowering were analyzed in subsequent generations.

### Screening of transgenic plants by PCR

Genomic DNA from leaves of T_0_ putative transformed plants as well as control plants was isolated using GenElute plant genomic DNA miniprep kit, according to the manufacturer’s instructions (Sigma, USA). PCR amplification of *cry1Ac* and *npt*II genes from plant genomic DNA (100 ng) was achieved by using set of primers (Additional file 5: Table S4) designed to amplify 995 bp, 768 bp and 678 bp amplicons of Tr*cry1Ac* and Fl*cry1Ac* and *npt*II genes respectively, in the GeneAmp® PCR system 9700 (PE Biosystems, USA). The 25 μl PCR reaction mixture was prepared containing 100 ng plant genomic DNA, 100 μM dNTPs mix, 25 ng of each primer, 2 mM MgSO_4_ and 1 U Taq DNA polymerase (NEB, USA). Amplification was performed with initial denaturation at 95°C for 5 min followed by 30 cycles, each comprising of denaturation at 94°C for 90 sec, annealing at 58°C (Tr*cry1Ac*)/67°C (Fl*cry1Ac*)/58°C (*npt*II) for 1 min and extension at 72°C for 3 min followed by final extension for 5 min at 72°C for 5 min. In all PCR experiments pRD400 plasmid was taken as positive control for *cry1Ac* gene and pNBRI–1 plasmid was taken as positive control for Fl*cry1Ac* gene. Amplified DNA fragments of PCR assays were electrophoresed on 1% agarose (w/v) gels, visualized, documented and analyzed on Gel Doc XR (Bio-Rad, USA).

### Southern blot hybridization analysis

Southern blot hybridization analysis was performed to confirm the integration of T-DNA into transformants according to Sambrook and Russell (2001), with few modifications (Koul et al. 2012). Aliquot of 10 μg genomic DNA purified from untransformed and transgenic plants was digested overnight with *Eco*RI (pRD400 transformants) and *Sal*I (pNBRI–1 transformants) cutting at single site within the T-DNA. The digested genomic DNA was separated by gel electrophoresis and transferred onto Bio Bond Plus nylon membrane (Sigma, USA). The blots were hybridized at 58°C for 24 h with 1,845 bp and 3,510 bp fragment of *cry1Ac* and FlCry1Ac genes respectively, radio labeled with αP^32^dCTP (BRIT, Mumbai India), washed under stringent conditions, exposed to Fuji screen for 48 h followed by scanning and documentation on Typhoon Trio Plus phosphoimager (GE Healthcare Life Sciences AB, Sweden).

### RT-PCR and quantitative real-time PCR

RT-PCR analysis of T_0_ and T_1_ transgenic plants was done by synthesis of first-strand of cDNA with enhanced Avian RT-PCR kit using 5 μg of total RNA purified from the transgenic plant according to manufacturer’s instructions (Sigma, USA). The relative quantity of *cry1Ac* transcripts in transgenic tomato plants was analyzed by quantitative PCR performed in StepOne real-time PCR system (Applied Biosystems, USA) using Quantifast SYBR green PCR kit (Qiagen, Germany). Tomato *β*-*actin* gene (GenBank accession no. U60482) was used as endogenous control in all real-time PCR assays. The nucleotide sequences of the set of primers for Tr*cry1Ac* gene were, forward; 5*′*-ACACAGTTTCTGCTCAGCGAGTT-3*′* and reverse; 5*′*- ACCAAAGATACCCCAGATGATGTC-3*′*, primers for Fl*cry1Ac* gene were, forward; 5*′*- CTTCTCTGGAACTGCTGGTGTGA-3*′* and reverse; 5*′*-CAGCCTTTTGGGCTCTTTCA-3*′*, while for *β*-*actin* gene forward; 5*′*-GCTGGATTTGCTGGAGATGATGA-3*′* and reverse; 5*′*-TCCATGTCATCCCAATTGCTAAC-3*′* giving an amplicon of 99, 101 and 194 bp respectively.

Total RNA extracted from 100 mg of leaf tissues was reverse transcribed into cDNA and used as template in real-time PCR assays with *cry1Ab* and *β*-*actin* gene-specific primers. Reverse transcription reaction was performed at 50°C for 10 min with initial denaturation at 95°C for 5 min (for activation of Hot-start Taq polymerase) followed by 40 amplification-cycles comprising of 10 s denaturation at 95°C and combined annealing and extension for 30 s at 60°C in 25 μl reaction mixture, according to manufacturer’s instructions (Qiagen, Germany). The relative values obtained from the quantitation of mRNA were expressed as 2^-ΔΔCt^ where ΔCt represents the difference between Ct (cycle threshold) values of a target and the endogenous control (*β*-*actin*) in the same sample and ΔΔCt is the difference between the ΔCt value of a particular sample and that of the reference sample. The quantitative data of real-time PCR represent mean values with standard error of three independent experiments with three replicates of the transgenic plant samples.

### Western immunoassay

Western immunoblotting of the transgenic plants expressing Cry1Ac toxin was performed using the cell-free extract of leaf tissues. Aliquots of the cell-free extracts were boiled for 10 min with 2 × sample loading dye (50 mM Tris–HCl pH 6.8, 100 mM DTT, 2% SDS, 0.1% bromophenol blue, 10% glycerol) and electrophoresed on 10% denaturing SDS-PAGE (Laemmli 1970). Protein bands were visualized following Coomassie blue staining and the other set of SDS-PAGE was transferred onto immunoblot™ PVDF membrane (Bio-Rad, USA) using trans-blot SD semi-dry transfer cell (Bio-Rad, USA) in transfer buffer (25 mM Tris base, 192 mM glycine, pH 8.3 and 0.1% SDS). The membrane was blocked for 2 h at 25°C in blocking buffer and incubated with primary antibody (rabbit polyclonal to Bt-Cry1Ac toxin, Amar Diagnostics, India) diluted to 1:1000 ratio in blocking buffer, for 2 h at 25°C. The membranes were washed four times with PBST, incubated in blocking buffer for 1 h followed by incubation with secondary antibody (goat polyclonal to rabbit IgG alkaline phosphatase conjugated antibody) at 1:5000 dilutions for 2 h at 25°C and developed with BCIP-NBT substrate solution (Sigma, USA).

### Quantitative estimation of Bt-toxin

Vegetative leaves from 12 weeks old transgenic tomato plants of T_0_ and T_1_ generations were used for protein extraction by grinding in 1:10 (w/v) ratio of plant tissue to PBST buffer (pH 7.4), in liquid nitrogen. The total soluble protein (TSP) concentration in cell-free extracts was determined by Bradford dye-binding procedure with bovine serum albumin (BSA) as standard protein (Bradford 1976). The quantitative estimation of expressed recombinant Cry1Ac (toxin) in cell-free extracts of transgenic plants was determined by double antibody sandwich enzyme-linked immunosorbent assay (DAS-ELISA), using peroxidase labeled PathoScreen kit for Bt-Cry1Ab/1Ac protein (Agdia, USA). Cell-free extracts (100 ng TSP) from leaves of transgenic plants were dispensed into wells of ELISA plate, pre-coated with primary antibody followed by reaction with secondary antibody conjugated with alkaline phosphatase to develop colour and detection of Cry1Ac toxin was monitored at 650 nm using SpectraMax 340PC spectrophotometer (Molecular Devices, USA).

### Insect bioassay

The larval populations of *H. armigera* were reared in the insectary on an artificial diet (Gupta et al. 2004) at 26 ± 2°C, 70% relative humidity on 14 h light and 10 h dark regime. The detached leaves from fourth to sixth nodes of untransformed control, T_0_ and T_1_ transgenic plants were washed thoroughly with distilled water, blotted dry and placed in a plastic container with 10 second instar larvae of *H. armigera* per leaf, in three replicates. Feeding was allowed for 48–96 h and the data on larval weight and percent mortality were analyzed statistically (SPSS Inc., USA). Each experiment was repeated thrice with three replicates and results were co-related to the quantitative expression of Cry1Ac toxin.

### Statistical analysis

Each experiment was performed with three replicates, unless otherwise mentioned and repeated at least three times. T_1_ seeds were germinated on MS basal medium supplemented with 50 mg l^−1^ kanamycin and subjected to χ^2^ fitness test for progeny segregation to compare the expected and observed data. All graphs were prepared using Sigma Plot software (Sigma Plot, USA).

## Additional files

Additional file 1: Table S1. Tomato transformation using pRD400 and pNBRI–1 for regeneration of transgenic plants. Additional file 2: Table S2. Insect mortality data of T_0_ transgenic plants. Additional file 3: Table S3. Segregation analysis of *npt*II gene in T_1_ seeds developed with vector pRD400 and pNBRI–1. Additional file 4: Figure S1. Quantitative assessment of Bt-Cry1Ab protein in different T_0_ plants and selected T_1_ population by A & B Double antibody sandwich enzyme-linked immunosorbent assay (DAS-ELISA) as percentage of total soluble protein (TSP) (*striped bar*) and corresponding insect mortality of *Helicoverpa armigera* (*open square*) and *Spodoptera litura* (*open circle*). Average quantity of Bt-Cry1Ab protein in transgenic plants is shown as % TSP ± standard deviation on the top of histogram bars and also indicated by horizontal mark for individual transgenic plant. ‘n’ is the number of T_1_ transgenic population from each parent. Additional file 5: Table S4. Sets of forward and reverse primers used in the experiments.

## Abbreviations

As: Acetosyringone
BAP: 6-Benzyladenine
BBMV: Brush bordered membrane vesicle
bp: Base pairs
cfu: Colony forming units
Cry: Crystal protein
DAS-ELISA: Double antibody sandwich enzyme-linked immunosorbent assay
*DECaMV35S*: *CaMV35S* promoter with duplicated enhancer
GA_3_: Gibberellic acid IAA: Indole-3-acetic acid
IBA: Indole-3-butyric acid
MS: Murashige and Skoog’s medium
*npt*II: Neomycin phosphotransferase
PBST: Phosphate buffered saline with Tween-20
PCR: Polymerase chain reaction
RIM: Root induction medium
SIM: Shoot induction medium
SEM: Shoot elongation medium
TSP: Total soluble protein
