# Supplementary material for: Comparative performance of modified full-length and truncated Bacillus thuringiensis-cry1Ac genes in transgenic tomato
Source: Springerplus. 2015 Apr 30;4:203. doi: 10.1186/s40064-015-0991-x (PMC4422829; doi:10.1186/s40064-015-0991-x)
Supplement: Additional file 1: Table S1. — Tomato transformation using pRD400 and pNBRI–1 for regeneration of transgenic plants. [file 40064_2015_991_MOESM1_ESM.doc]

**Additional file 1: Table S1** Tomato transformation using pRD400 and pNBRI–1 for regeneration of transgenic plants

|  | I^st^ selection cycle | | | | II^nd^ successive selection cycle | | | | | | | | | | | | | | | |
| --- | --- | --- | --- | --- | --- | --- | --- | --- | --- | --- | --- | --- | --- | --- | --- | --- | --- | --- | --- | --- |
| Vector (gene) | Number of  explants used (A ) | | Number of responding  explants^a^ (B) | | % response^b^ (B/A) | | Number of shoot  buds produced | | Number of explants  inoculated (C) | | Number of responding  explants (D) | | | % response (D/C) | | | Antibiotic resistant  plants produced (E) | | % frequency of  transformation (E/A) | |
| pRD400 (cry1Ac) | 170 | 592 | 64 | 263 | 35.16 | 40.98 | 48 | 240 | 310 | 1006 | | 63 | 211 | | 20.32 | 21.65 | 26 | 99 | 15.29 | 16.93 ± 2.10 |
|  | 142 |  | 59 |  | 41.25 |  | 55 |  | 285 |  |  | 48 |  |  | 16.84 |  | 23 |  | 16.42 |  |
|  | 150 |  | 68 |  | 39.53 |  | 73 |  | 175 |  |  | 48 |  |  | 27.43 |  | 24 |  | 16.00 |  |
|  | 130 |  | 72 |  | 48.00 |  | 64 |  | 236 |  |  | 52 |  |  | 22.03 |  | 26 |  | 20.00 |  |
| pNBRI1 (Flcry1Ac) | 115 | 469 | 32 | 154 | 27.83 | 32.64 | 40 | 232 | 106 | 447 | | 19 | 66 | | 17.92 | 14.92 | 9 | 44 | 7.80 | 9.30 ± 2.04 |
|  | 120 |  | 52 |  | 43.33 |  | 89 |  | 139 |  |  | 17 |  |  | 12.23 |  | 13 |  | 10.80 |  |
|  | 124 |  | 41 |  | 33.06 |  | 55 |  | 114 |  |  | 17 |  |  | 14.91 |  | 14 |  | 11.30 |  |
|  | 110 |  | 29 |  | 26.36 |  | 48 |  | 88 |  |  | 13 |  |  | 14.62 |  | 8 |  | 7.30 |  |
| +Control^c^ | 48 | 178 | 47 | 167 | 97.92 | 93.82 | 118 | 443 | 236 | 787 | | 233 | 765 | | 98.72 | 97.02 | – | | – | |
|  | 44 |  | 40 |  | 90.90 |  | 108 |  | 194 |  |  | 191 |  |  | 98.45 |  | – | | – | |
|  | 46 |  | 44 |  | 95.65 |  | 112 |  | 158 |  |  | 150 |  |  | 94.94 |  | – | | – | |
|  | 40 |  | 36 |  | 90.00 |  | 105 |  | 199 |  |  | 191 |  |  | 95.98 |  | – | | – | |
| –Control^d^ | 52 | | 8 | | 15.30 | | 0 | | – | | – | | | – | | | – | | – | |

^a^The explants which regenerate during selection cycle (explants having kanamycin resistance gene).

^b^The values written before the braces for the column % response are the average values, otherwise it represents the sum of the individual values of four experiments.

^c^Control tomato leaf explant kept on tomato tissue culture medium devoid of antibiotics.

^d^Control tomato leaf explant kept on tomato tissue culture medium and subjected to antibiotic selection regime.
