# Supplementary material for: Comparative performance of modified full-length and truncated Bacillus thuringiensis-cry1Ac genes in transgenic tomato
Source: Springerplus. 2015 Apr 30;4:203. doi: 10.1186/s40064-015-0991-x (PMC4422829; doi:10.1186/s40064-015-0991-x)
Supplement: Additional file 2: Table S2. — Insect mortality data of T0 transgenic plants. [file 40064_2015_991_MOESM2_ESM.doc]

| **T_0_ transgenic plant ID** | **Cry1Ac toxin**  **(% of TSP)** | **% mortality**  **(±10)** | **% wt loss** | **T_0_ transgenic plant ID** | **Cry1Ac toxin**  **(% of TSP)** | **% mortality**  **(±10)** | **% wt loss** |
| --- | --- | --- | --- | --- | --- | --- | --- |
| **Control**  Ac1 | -  0.0058 | 0  70 | 0  55 ± 0.135 | **Control**  FLAc1 | -  0.0008 | 0  30 | 0  36 ± 0.117 |
| Ac2 | 0.0069 | 70 | 60 ± 0.082 | FLAc2 | 0.0011 | 50 | 55 ± 0.232 |
| Ac3 | 0.0032 | 50 | 24 ± 0.117 | FLAc3 | 0.0009 | 30 | 39 ± 0.099 |
| Ac4 | 0.0042 | 70 | 52 ± 0.123 | FLAc4 | 0.0008 | 30 | 37 ± 0.125 |
| Ac5 | 0.0083 | 80 | 74 ± 0.070 | FLAc5 | 0.0005 | 20 | 29 ± 0.110 |
| Ac6 | 0.0074 | 80 | 71 ± 0.088 | FLAc6 | 0.0006 | 30 | 34 ± 0.084 |
| Ac7 | 0.0120 | 90 | 79 ± 0.057 | **FLAc7** | **0.0015** | **100** | ***** |
| Ac8 | 0.0056 | 70 | 57 ± 0.116 | FLAc8 | 0.0003 | 30 | 23 ± 0.048 |
| **Ac9** | **0.0240** | **100** | ***** | FLAc9 | 0.0001 | 20 | 19 ± 0.057 |
| Ac10 | 0.0029 | 50 | 23 ± 0.095 | FLAc10 | 0.0009 | 40 | 42 ± 0.114 |
| **Ac11** | **0.0128** | **90** | **82 ± 0.063** | **FLAc11** | **0.0026** | **100** | ***** |
| Ac12 | 0.0120 | 90 | 81 ± 0.057 | FLAc12 | 0.0004 | 30 | 23 ± 0.048 |
| Ac13 | 0.0045 | 70 | 54 ± 0.117 | FLAc13 | 0.0001 | 20 | 15 ± 0.053 |
| Ac14 | 0.0067 | 80 | 64 ± 0.126 | FLAc14 | 0.0002 | 20 | 16 ± 0.052 |
| Ac15 | 0.0160 | 90 | 84 ± 0.052 | FLAc15 | 0.0006 | 30 | 29 ± 0.099 |
| **Ac16** | **0.0380** | **100** | ***** | FLAc16 | 0.0009 | 40 | 40 ± 0.105 |
| Ac17 | 0.0038 | 50 | 31 ± 0.088 | FLAc17 | 0.0008 | 30 | 38 ± 114 |
| Ac18 | 0.0168 | 90 | 85 ± 0.053 | FLAc18 | 0.0002 | 10 | 15 ± 0.071 |
| Ac19 | 0.0174 | 90 | 86 ± 0.052 | FLAc19 | 0.0004 | 20 | 23 ± 0.067 |
| Ac20 | 0.0160 | 80 | 83 ± 0.067 | FLAc20 | 0.0009 | 30 | 40 ± 0.115 |
| **Ac21** | **0.0255** | **100** | ***** | FLAc21 | 0.0006 | 30 | 31 ± 0.110 |
| Ac22 | 0.0020 | 40 | 18 ± 0.063 | FLAc22 | 0.0009 | 40 | 42 ± 0.114 |
| Ac23 | 0.0145 | 90 | 78 ± 0.042 | FLAc23 | 0.0003 | 20 | 21 ± 0.063 |
| Ac24 | 0.0048 | 70 | 80 ± 0.047 | FLAc24 | 0.0010 | 40 | 44 ± 0.107 |
| **Ac25** | **0.0386** | **100** | ***** | FLAc25 | 0.0010 | 50 | 46 ± 0.117 |
| **Ac26** | **0.1259** | **100** | ***** | FLAc26 | 0.0004 | 20 | 26 ± 0.084 |
| Ac27 | 0.0086 | 80 | 77 ± 0.048 | FLAc27 | 0.0008 | 20 | 33 ± 0.125 |
| Ac28 | 0.0080 | 80 | 72 ± 0.079 | FLAc28 | 0.0003 | 10 | 18 ± 0.042 |
| Ac29 | 0.0174 | 90 | 87 ± 0.048 | FLAc29 | 0.0009 | 30 | 38 ± 0.123 |
| Ac30 | 0.0024 | 60 | 21 ± 0.074 | FLAc30 | 0.0005 | 20 | 30 ± 0.115 |

**Additional file 2: Table S2 Insect mortality data of T_0_ transgenic plants**

*100 % mortality; Control : Non-trangenic tomato plant
