# Supplementary material for: Comparative performance of modified full-length and truncated Bacillus thuringiensis-cry1Ac genes in transgenic tomato
Source: Springerplus. 2015 Apr 30;4:203. doi: 10.1186/s40064-015-0991-x (PMC4422829; doi:10.1186/s40064-015-0991-x)
Supplement: Additional file 3: Table S3. — Segregation analysis of nptII gene in T1 seeds developed with vector pRD400 and pNBRI–1. [file 40064_2015_991_MOESM3_ESM.doc]

**Additional file 3: Table S3** **Segregation analysis of *npt*II gene in T_1_ seeds developed with vector pRD400 and pNBRI–1**

| **T_0_ transgenic plants** | **Response of seeds on kanamycin selection medium** | | | **T_0_ transgenic plants** | **Response of seeds on kanamycin selection medium** | | |
| --- | --- | --- | --- | --- | --- | --- | --- |
| **Plant ID** | **Total** | **Kan^r^ /Kan^s^** | **χ^2^ value^a^** | **Plant ID** | **Total** | **Kan^r^ /Kan^s^** | **χ^2^ value^a^** |
| TrAc 9 | 24 | 16/8 | 0.89 | FLAc 7 | 86 | 60/26 | 1.25 |
| TrAc 16 | 76 | 64/12 | 3.34 | FLAc 11 | 91 | 72/19 | 0.82 |
| TrAc 21 | 50 | 43/7 | 3.22 |  |  |  |  |
| TrAc 25 | 28 | 20/8 | 0.19 |  |  |  |  |
| TrAc 26 | 30 | 18/12 | 1.17 |  |  |  |  |

^a^χ^2^_1_ = 3.841 at ≤ 0.05. *Kan^r^* Kanamycin-resistant, *Kan^s^* Kanamycin-sensitive.
