# Supplementary figures and images for: Comparative performance of modified full-length and truncated Bacillus thuringiensis-cry1Ac genes in transgenic tomato
Source: Springerplus. 2015 Apr 30;4:203. doi: 10.1186/s40064-015-0991-x (PMC4422829; doi:10.1186/s40064-015-0991-x)

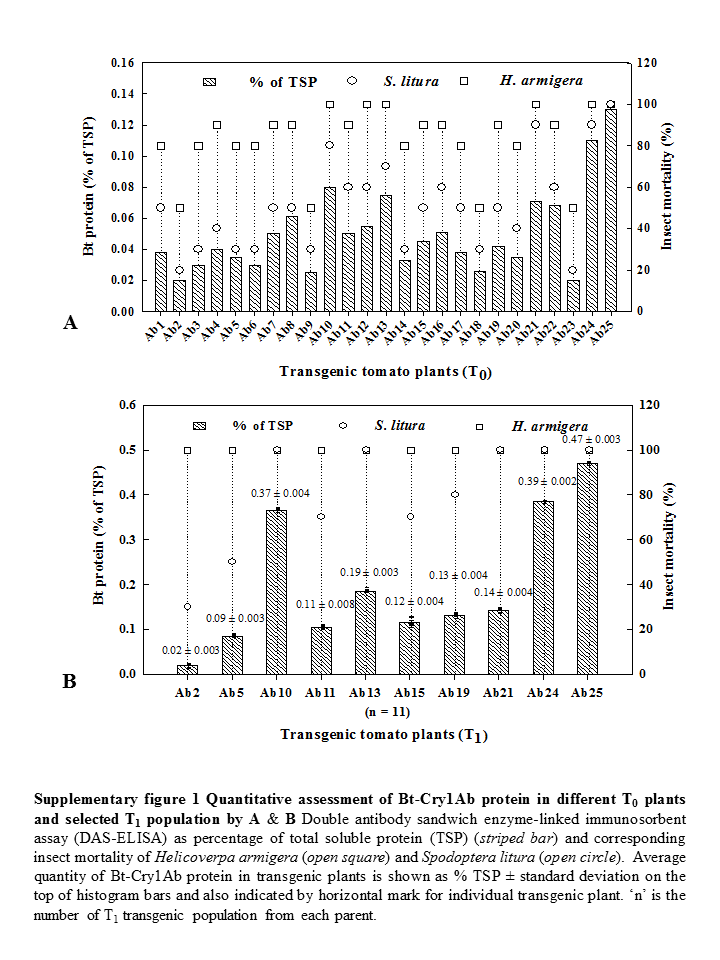

Supplement: Additional file 4: Figure S1. — Quantitative assessment of Bt-Cry1Ab protein in different T0 plants and selected T1 population by A & B Double antibody sandwich enzyme-linked immunosorbent assay (DAS-ELISA) as percentage of total soluble protein (TSP) (striped bar) and corresponding insect mortality of Helicoverpa armigera (open square) and Spodoptera litura (open circle). Average quantity of Bt-Cry1Ab protein in transgenic plants is shown as % TSP ± standard deviation on the top of histogram bars and also indicated by horizontal mark for individual transgenic plant. ‘n’ is the number of T1 transgenic population from each parent. [file 40064_2015_991_MOESM4_ESM.png]
