# Supplementary material for: Comparative performance of modified full-length and truncated Bacillus thuringiensis-cry1Ac genes in transgenic tomato
Source: Springerplus. 2015 Apr 30;4:203. doi: 10.1186/s40064-015-0991-x (PMC4422829; doi:10.1186/s40064-015-0991-x)
Supplement: Additional file 5: Table S4. — Sets of forward and reverse primers used in the experiments. [file 40064_2015_991_MOESM5_ESM.doc]

**Additional file 5: Table S4 Sets of forward and reverse primers used in the experiments**

| **Primers** | **Gene size**  **(bp)** | **Sequence of primers (5′–3′)** | **Anealing temperature (ºC)** | **Amplicon size (bp)** |
| --- | --- | --- | --- | --- |
| A1 (*npt*II-forward) | 1,635 | TATTCGGCTATGACTTGGGC | 58 | 678 |
| B1 (*npt*II-reverse) |  | GCGAACGCTATGTCCTGATA |  |  |
| A2 (*cry1Ac*-forward) | 1,845 | ATTCCTGGTGCAAATTGAGC | 58 | 995 |
| B2 (*cry1Ac*-reverse) |  | CGATTCCGCTCTTTCTGTAA |  |  |
| A3 (Fl*cry1Ac*-forward) | 3,510 | CAGAGGTGGGGATTCGATGCTG | 67 | 768 |
| B3 (Fl*cry1Ac*-reverse) |  | CATTGACACGTGGGACAATCGAT |  |  |
